# Under-Reporting of Road Traffic Mortality in Developing Countries: Application of a Capture-Recapture Statistical Model to Refine Mortality Estimates

**DOI:** 10.1371/journal.pone.0031091

**Published:** 2012-02-15

**Authors:** Jonathan C. Samuel, Edward Sankhulani, Javeria S. Qureshi, Paul Baloyi, Charles Thupi, Clara N. Lee, William C. Miller, Bruce A. Cairns, Anthony G. Charles

**Affiliations:** 1 Department of Surgery, Kamuzu Central Hospital, Lilongwe, Malawi; 2 Department of Surgery, School of Medicine, University of North Carolina, Chapel Hill, Chapel Hill, North Carolina, United States of America; 3 National Road Safety Council, Lilongwe, Malawi; 4 Department of Epidemiology, School of Public Health, University of North Carolina, Chapel Hill, Chapel Hill, North Carolina, United States of America; 5 Department of Medicine, School of Medicine, University of North Carolina, Chapel Hill, Chapel Hill, North Carolina, United States of America; 6 North Carolina Jaycee Burn Center, University of North Carolina Chapel Hill, Chapel Hill, North Carolina, United States of America; INSERM & Universite Pierre et Marie Curie, France

## Abstract

Road traffic injuries are a major cause of preventable death in sub-Saharan Africa. Accurate epidemiologic data are scarce and under-reporting from primary data sources is common. Our objectives were to estimate the incidence of road traffic deaths in Malawi using capture-recapture statistical analysis and determine what future efforts will best improve upon this estimate. Our capture-recapture model combined primary data from both police and hospital-based registries over a one year period (July 2008 to June 2009). The mortality incidences from the primary data sources were 0.075 and 0.051 deaths/1000 person-years, respectively. Using capture-recapture analysis, the combined incidence of road traffic deaths ranged 0.192–0.209 deaths/1000 person-years. Additionally, police data were more likely to include victims who were male, drivers or pedestrians, and victims from incidents with greater than one vehicle involved. We concluded that capture-recapture analysis is a good tool to estimate the incidence of road traffic deaths, and that capture-recapture analysis overcomes limitations of incomplete data sources. The World Health Organization estimated incidence of road traffic deaths for Malawi utilizing a binomial regression model and survey data and found a similar estimate despite strikingly different methods, suggesting both approaches are valid. Further research should seek to improve capture-recapture data through utilization of more than two data sources and improving accuracy of matches by minimizing missing data, application of geographic information systems, and use of names and civil registration numbers if available.

## Introduction

Road traffic injuries (RTIs) are a significant cause of preventable death and disability in developing countries, including those in Africa [1]. The WHO estimates that every year, RTIs in low and middle income African countries accounts for 168 000 deaths and 5.8 million lost disability-adjusted life years [2].

Despite the recognition of the severity of the problem, accurate data on RTIs in developing countries are scarce and incomplete [3]. Malawi lacks an organized trauma care system of prompt communication and trauma system activation, prompt system response, and the administrative documentation of episodes of care which is required for system review and public health intervention [4]. There is no equivalent to the National Trauma Data Base, Medicare records, or other clinical data sets which facilitate an understanding of injury epidemiology in the United States [5]. Given this lack of epidemiologic data in Malawi and the perceived magnitude of the problem, an accurate means of estimating RTIs is critical. This is important not only for injury surveillance, but for developing primary (or “pre-event” such as pedestrian facilities), secondary (or “event” such as helmet laws), and tertiary (or “post-event” such as hospital trauma care) injury prevention efforts [6]. Additionally, monitoring and evaluation of such interventions requires accurate epidemiologic data.

Two-source capture-recapture analysis has been used to estimate road traffic injuries and deaths in other regions of the world were data sources are incomplete [7], [8], [9], [10]. To our knowledge, however, this method has not previously been employed in low and middle income Africa where data is disproportionately lacking compared to developed regions, and hence capture-recapture analysis might be a particularly appropriate statistical model. Additionally, previous efforts have not accounted for both heterogeneity and stringency of matching criteria, two factors which bias capture-recapture results [11]. We sought to account for both of these biases while applying capture-recapture analysis to data on road traffic injuries and deaths in the Lilongwe district of Malawi.

The primary objective of this study was to determine an accurate estimate of the incidence of road traffic injuries and fatalities in Malawi using hospital registry and police data, and apply a two-source capture-recapture analysis of available data. The secondary objective was to determine how to improve future estimations by addressing inherent methodological issues and by practical considerations related to the study environment.

## Materials and Methods

### Hospital registry and police accident data sources

This study utilized one year of data (July 2008 to June 2009) from the trauma registry data at Kamuzu Central Hospital (KCH) in Lilongwe, Malawi and police accident report data from the National Road Safety Council (NRSC) of Malawi [12]. This study was approved as a retrospective data analysis with a waiver of consent by both the Institutional Review Board of the University of North Carolina and the National Health Sciences Review Committee of Malawi. KCH, a 1000 bed hospital, is the only tertiary referral center for the entire central region of Malawi (population five million) thus serving as a the most central and specialized source of care after smaller district hospitals and local health centers. The hospital registry includes data on all patients presenting to KCH for treatment of injuries. Data are collected from the patients and those accompanying the patients at all times by dedicated clerks, and includes victim demographics, pre-hospital information including time, location, and mechanism of injury, mode of transport to the hospital, and admitting vital signs, injuries, and diagnoses. The subset of patients in the hospital registry having died (dead on arrival, died in casualty department, or died prior to discharge) from RTIs within the Lilongwe district was selected for analysis. The NRSC manages data gathered by the police on road traffic collisions. These police data are collected throughout Malawi on road-related collisions reported to the police, regardless of whether injuries were sustained. Police data include incident-related factors (including accident type and vehicle(s) involved, and environmental factors such as road geometry and hazards, and time of day) and detailed information pertaining to those persons involved (including number injured or killed, license and insurance status, and behavioral factors). The subset of police data analyzed in this study included all incidents within the Lilongwe district in which at least one person died. For the purposes of this analysis, the variables used from and present in both of the data sources were age, gender, road user type, number of injuries and vehicles involved in the incident, date and time of day of incident, and incident location.

### Data matching

The subsets of data described above, representing RTIs in the Lilongwe district, were compared, and persons included in both sources were identified as a match. A match was made when gender, age (within five years), injury mechanism, location, and time (within three hours) matched, with one missing variable allowed as long as the other parameters were met. For location matching, data reviewers familiar with the Lilongwe district reviewed the reported village, street, and landmark data and determined whether the location was a match as described previously [9].

### Simple capture-recapture analysis

To perform capture-recapture analysis, the estimated number of events (*ñ*), variance of *ñ* (var(*ñ*) ), and 95% confidence intervals (95% CI) were calculated based on the number of events recorded in the trauma registry, *d_1_*, the number of events recorded in the police accident reports, *d_2_*, and the number of events reported in both the trauma registry and police accident reports, *m*:
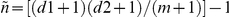


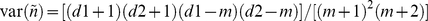


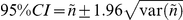
A simple capture-recapture analysis was performed using the above formulas.

### Stratified capture-recapture analysis

Chi-squared tests were performed to test independence between the hospital and police data for the following factors: gender (male, female or unreported); road user type (pedestrian, bicyclist, motorcyclist, driver, passenger, or unreported); number of injured and killed involved per incident (one or two versus ≥three); and number of vehicles involved per incident (zero or one versus ≥two). For each of these factors, a stratified capture-recapture analysis was done by performing capture-recapture analyses on the subgroups, and then summing the subgroup results to derive an overall estimate. For these stratified analyses, confidence intervals for stratified estimates were calculated using Monte Carlo analysis as reported previously [13].

### Incidence calculations

Using the various estimates for deaths, incidence was calculated using 2008 Malawi census data [14]. These incidence estimates were then compared to World Health Organization estimates for road traffic deaths in Malawi, and in the low and middle income African region [15].

## Results

### Comparison of data sources

The hospital registry and police accident data identified 97 and 143 fatalities, respectively (Table 1). In both data sources, most victims tended to be male, and the most common road users injured were pedestrians. The main differences identified between the two data sources were that the police accident data, compared to hospital registry, tended to capture more males, more pedestrian and driver victims, more incidents with multiple vehicles, and fewer passenger victims (p<0.001 for each using Chi-Squared tests of inequality). There were 36 matches in the dataset (Table 1).

**Table 1:** Capture-Recapture Estimates of Deaths.

Stratified by		Number of Matches	Number unmatched in police data	Number unmatched in hospital data	Est. Number of Events (95%CI)	Est. Incidence (95%CI) (per 1000 person-years)
	total	36	107	61	380 (298–463)	0.200 (0.157–0.244)
Gender	male	31	93	42	288 (224–352)	
	female	5	12	16	65 (31–99)	
	missing	0	2	3	11 (3–23)	
	total	36	107	61	364 (290–438)	0.192 (0.153–0.231)
Road User	pedestrian	18	59	26	184 (130–238)	
	bicyclist	8	17	5	39 (27–51)	
	motorcyclist	1	0	1	2 (2–2)	
	driver	4	16	3	33 (19–47)	
	passenger	4	14	23	105 (39–171)	
	other/missing	1	1	3	7 (3–11)	
	total	36	107	61	370 (283–457)	0.195 (0.149–0.241)
Victims/Incident	1–2	28	87	34	251 (194–308)	
	≥3	8	20	27	115 (63–167)	
	total	36	107	61	366 (289–443)	0.193 (0.153–0.234)
Vehicles/Incident	0–1	33	86	47	285 (224–346)	
	≥2	3	21	14	112 (32–192)	
	total	36	107	61	396 (296–497)	0.209 (0.156–0.262)

### Incidence calculations

The incidences of road traffic deaths as calculated from registered deaths in the police accident and hospital registry data were 0.075 and 0.051 deaths/1000 person-years, respectively. Simple and stratified capture-recapture analyses revealed mortality incidence estimates that ranged from 0.192 to 0.209 deaths/1000 person-years (Table 1). A comparison of capture-recapture estimates for road traffic deaths to the WHO estimates for Malawi and the low-middle income African region revealed that our estimates were similar to the WHO estimate for Malawi (0.236 deaths/1000 person-years) [15].

### Missing Data

There were no missing values in the police data. The hospital registry had missing data for age (N = 46), time (N = 7), and location (N = 4). Overall there were a total of 53 mortalities that had missing data, but only 3 had more than one variable missing (age/time N = 2, age/time/location N = 1). The majority of deaths recorded in the hospital were victims who were dead on arrival (82 of 97 deaths, data not shown), and many were unidentified meaning age was not available.

## Discussion

### Implications of study findings

Capture-recapture analysis is a powerful tool to estimate the incidence of road traffic deaths using two incomplete data sources. Using police accident reports and a hospital-based trauma registry we estimated the incidences of RTI deaths in the Lilongwe district of Malawi. These estimates using capture-recapture analyses were much higher than that of either data source alone.

Using capture-recapture analysis, our estimate of RTI deaths (0.192 deaths/1000 person-years) was slightly lower than the WHO estimate for Malawi (0.236 deaths/1000 person-years) [15]. The WHO estimated mortality by first gathering data from countries with very detailed information on causes of death (defined as reporting >85% of country-wide deaths, with <30% of these recorded as unknown cause of death) , which corresponded to 37 high-income and 38 low to middle-income countries. Next this data was standardized to a 30-day incidence using the European Conference of Ministers of Transport correction factors. A regression model was then derived from data from these countries, with the dependent variable being 30-day mortality, and the independent variables being income (GNI), income level, population, vehicle density, road density, existence of a national helmet law, national policies encouraging walking and cycling, or investment in public transport, urban and rural speed limits, alcohol consumption, and strength of the health system. These variables were selected through a literature review conducted by the WHO. This regression model was then used to estimate road traffic mortality in the remaining 3 high-income and 91 low to middle-income countries with less complete data (including Malawi) [15], [16].

### Environmental limitations

We used relatively relaxed matching criteria; Tercero *et al.* used similar criteria to ours, but required time to match within one hour and did not allow for missing data as we did [10]. In Malawi, as in many other developing countries, there are inherent difficulties in interpreting injury surveillance data. Often persons do not know their age, and it is estimated by either themselves or the data collector. In addition, time and location are also prone to being inaccurate. In traditional culture in Malawi, time of day does not have the same significance as it does in many Western societies. Furthermore, pre-hospital transport time is much longer in our population than in developed countries [12]. Both these factors introduce significant recall bias when persons are queried as to the time of the incident. Lastly, location requires careful analysis by persons knowledgeable with the area of interest which is prone to user variability and lacks the quantitative rigor of techniques such as geographic information systems (GIS) analysis using global positioning system coordinates (which are not available in the police or hospital data sets). To address the challenges in matching introduced by missing and inaccurate data we used relatively relaxed matching criteria which if biased would tend to lower our estimate (due to the increased potential for false matches which would result in a falsely increased data set overlap).

The WHO estimate is in close agreement with our results suggesting both methods though quite different are accurate. To reduce the bias introduced by difficulties with matching, police data could include the victim name in addition to age and gender, which would assist greatly in matching. In the future, civil identification numbers may also be available in Malawi which would also improve matching accuracy.

The overall effect of missing data was addressed by allowing our matching criteria to define a match with one missing data point. The effect of this on our estimation was to identify matches more frequently, therefore lowering the estimated number of deaths. This approach was required because the majority of deaths recorded in the hospital registry were victims who were dead on arrival (82 of 97 deaths), and many were unidentified meaning age was not available. If our matching criteria had required age to match, these unidentified victims would have by definition never matched to police data which would lead to an erroneously high estimation. Indeed, as a sensitivity analysis we conducted a capture-recapture analysis with a matching algorithm that required age to match, and as expected found that our estimated mortality incidence was 0.448–0.592/1000 person-years (data not shown), which essentially validated to need to allow for missing data due to the high number of unidentified victims in the hospital data. Future hospital-based data collection might limit this effect by integrating mortuary data which often includes age, as it becomes known when bodies are claimed by family [17].

### Methodological limitations

There are several methodological biases of capture-recapture which also must be addressed to ensure accuracy of results. The fist bias is that of heterogeneity, or the situation that arises when there is an unequal likelihood of capture between the two data sources. Stated more conceptually, the two data sources must be independent; the average product of the probability of being captured in either data source should equal the average probability of being captured by both sources [18].

Because our data sources are not independent random samples, we addressed this bias with stratified capture-recapture: using chi-squared analyses we identified several factors that were associated with dependency of the data sources; gender, road user type, number of victims per incident and number of vehicles per incident. Since these factors were associated with dependency between data sources, we applied a stratified capture-recapture analyses to control for this bias. The results indicated that heterogeneity did influence results, but to a much lesser degree than did the choice of matching criteria.

It is unclear exactly why police data compared to the hospital registry tended to disproportionately capture males, pedestrians and drivers (versus bicyclists) and multi-vehicle incidents. One possible explanation is that police reports are more likely to be filed when incidents involve relatively more injuries, deaths, and property damage. This potential bias has implications for future research. For example utilizing insurance claims in future capture-recapture analyses might lead to more matches to police data than to hospital data which could significantly affect heterogeneity.

### Recommendations

To better refine estimates, it would be helpful to use additional data sources, as has been done previously to study adolescent injuries [19]. One potential source in Malawi is mortuary data from the other district hospitals and health centers throughout Malawi. Another means to refine estimates would be to apply spatial analyses to the data, which would not only control for geographic heterogeneity between sources, but could also reveal unique patterns of injury relevant to prevention [20].

In conclusion, capture-recapture analysis of road traffic deaths in a developing country such as Malawi provides a means to overcome the limitations of incomplete data sources. Further research, such as using a third data source, or applying spatial analyses, will refine such estimates. We recommend using capture-recapture analysis in other locations where estimates of RTI injuries and deaths are incomplete, especially in developing countries where the burden of injury from RTIs is high. Care must be taken, however, to control for source heterogeneity (sources which are not random and independent) and to address practical considerations related to the choice of matching criteria.
